# CXCL9 and CXCL10 display an age-dependent profile in Chagas patients: a cohort study of aging in Bambui, Brazil

**DOI:** 10.1186/s40249-020-00663-w

**Published:** 2020-05-11

**Authors:** Fernanda Fortes de Araújo, Karen Cecília Lima Torres, Sérgio Viana Peixoto, Antonio Luiz Pinho Ribeiro, Juliana Vaz Melo Mambrini, Vitor Bortolo Rezende, Maria Luiza Lima Silva, Antônio Ignácio Loyola Filho, Andréa Teixeira-Carvalho, Maria Fernanda Lima-Costa, Olindo Assis Martins-Filho

**Affiliations:** 1 Integrated Research Group in Biomarkers, Rene Rachou Institute, Oswaldo Cruz Foundation, Avenida Augusto de Lima, 1715 – Barro Preto –, Belo Horizonte, Minas Gerais 30190-002 Brazil; 2 José do Rosário Vellano University, UNIFENAS/BH, Belo Horizonte, Brazil; 3 Center for Studies in Public Health and Aging, Rene Rachou Institute, Oswaldo Cruz Foundation, Belo Horizonte, Brazil; 4 Clinical Hospital and Faculty of Medicine, Federal University of Minas Gerais, Belo Horizonte, Brazil

**Keywords:** Chagas disease, Immune biomarkers, Chemokines, Cytokines, Cohort

## Abstract

**Background:**

Chagas disease is endemic in Latin America and still represents an important public health problem in the region. Chronic cardiomyopathy is the most significant chronic form due to its association with morbidity and mortality. The last decade has seen increasing evidence that inflammatory cytokines and chemokines are responsible for the generation of inflammatory infiltrate and tissue damage, with chronic chagasic cardiomyopathy patients presenting a pro-inflammatory immune response. Although studies have evaluated the role of chemokines in experimental *T. cruzi* infection, few have addressed their systemic profile, especially for human infection and in aging populations. The present work aimed to use the data from a large population based study of older adults, conducted in an endemic area for Chagas disease, to examine the association between serum levels of cytokines and chemokines, *T. cruzi* infection and electrocardiogram (ECG) abnormality.

**Methods:**

The present work evaluated serum levels of CCL2, CXCL9, CXCL10, CCL5, CXCL8, IL-1β, IL-6, TNF, IL-12 and IL-10 by Flow Cytometric Bead Array assay (CBA) and the results expressed in pg/ml. The baseline survey started in January 1st 1997, with 1284 participants of an aged population-based cohort. Participants signed an informed consent at baseline and at each subsequent visit and authorized death certificate and medical records verification.

**Results:**

Our results demonstrated that Chagas disease patients had higher serum levels of CXCL9, CXCL10 and IL-1β and lower serum levels of CCL5 than non-infected subjects. Moreover, our data demonstrated that CXCL9 and CXCL10 increased in an age-dependent profile in Chagas disease patients.

**Conclusion:**

Together, this study provided evidences that serum biomarkers increase along the age continuum and may have potential implications for establishing clinical management protocols and therapeutic intervention in Chagas disease patients.

## Background

Chagas disease (CD) or American trypanosomiasis is an infection caused by *Trypanosoma cruzi*, a protozoan that is endemic in Latin America [1]. The disease has spread throughout the world, but especially in the United States, Europe, Asia and Oceania due to international immigration [2]. There are estimates that about 8 million people are currently infected with *T. cruzi,* mainly in Latin America, with risk of developing chronic forms of the disease [3].

There are important knowledge gaps about the natural history of CD and its clinical course is highly variable. Most infected individuals develop the chronic phase of the disease and many remain asymptomatic throughout their lifetime, in the indeterminate form of the disease, while 20 to 40% develop cardiac or digestive forms [1, 3]. Cardiac cardiomyopathy is the most important chronic form of Chagas disease, because of its association with morbidity and mortality and the consequent medical and social impact [4]. Infected patients with the chronic indeterminate form evolve to Chagas cardiomyopathy at a rate of 2% annually [5], but which patients and why they develop heart diseases (and others do not) is largely unknown.

The mechanisms involved on the development of severe forms of Chagas disease are not well defined. However, previous studies have demonstrated the importance of immune response in disease progression and that the balance between inflammatory and anti-inflammatory cytokines produced by circulating cells in patients with indeterminate clinical form leans towards an anti-inflammatory profile, contributing to the control of the disease in these patients. On the other hand, cardiac patients developed a Th1-specific immune response that is associated with morbidity [6–10].

The last decade has seen increasing evidence that inflammatory cytokines and chemokines are responsible for the generation of inflammatory infiltrate and tissue damage [11]. Previous studies have demonstrated that mononuclear cells infiltrating CCC heart tissue express IFN-γ, TNF and IL-6, with lower levels of IL-2, IL-4 and IL-10 [12–14]. Moreover, specific chemokines are produced in tissue in response to *T. cruzi* infection and are crucial in defining leukocyte subtypes that compose the inflammatory infiltrate in the heart of infected animals [15–17]. Furthermore, Cunha-Neto et al., [18] detected mononuclear cells that express CXCR3, CCR5, CXCL9 and CCL5 in the myocardium of CCC patients using confocal immunofluorescence assays. These authors suggested that IFN-γ-dependent chemokines, such as CCL5, CXCL9 and CXCL10, may increase the chemotactic signal and cause migration of more T lymphocytes to the myocardium. Although studies have evaluated the role of chemokines in experimental *T. cruzi* infection, few studies have addressed their profile, especially in human infection.

We used data from a large population based study of older adults conducted in and endemic area for Chagas disease to examine the association between serum levels of cytokines and chemokines, *T. cruzi* infection and electrocardiogram (ECG) abnormality, taking into account an array of potential confounding variables.

## Methods

### Study design and population

The cohort study of aging is ongoing in Bambuí, a city of approximately 15 000 inhabitants in the state of Minas Gerais in Southeast Brazil, which is one of the oldest known endemic areas for Chagas disease [19–21]. Detailed information on this cohort can be found elsewhere [22]. Briefly, a cohort baseline survey was conducted in 1997 and the study population was identified by a complete census in the city. Participants signed an informed consent at baseline and at each subsequent visit and authorized death certificate and medical records verification. All residents aged 60 or older (1742) were invited to join the study; 1496 (85.9%) authorized blood sample tests.

The Bambui cohort included in this study presented an overall seroprevalence of 37.5% for *T. cruzi* infection, including 35.6% for patients aged 60 to 69 years old, and 40.5% for both patients aged 70 to 79 and 80+ years old. According to previous studies carried out in the area of Bambui city, TcII is the predominant genotype being found in approximately 77.6% of *T. cruzi* infected patients [23].

This study was approved by the Ethics Research Committee at Fundação Oswaldo Cruz (CAAE 01082212.7.0000.5091). All participants signed an informed consent form for all procedures.

### Chagas disease serology

Serology for *T. cruzi* infection was assessed by three different assays performed concurrently: a hemagglutination assay (Biolab Merieux SA, Rio de Janeiro, Brazil) and two enzyme- linked immunosorbent assays (Abbott Laboratories, Inc., North Chicago, Illinois; and Wiener Laboratories, Rosario, Argentina). Infection with *T. cruzi* was defined by seropositivity in all three of the assays; seventeen persons had discordant results among the assays and were excluded from the analysis.

### Electrocardiogram (ECG)

At the baseline examination, a digitally recorded 12-lead ECG (Hewlett Packard MI700A) reading was obtained at rest. The ECGs were analyzed at the ECG Reading Center (EPICARE, Wake Forest University) and classified using the Minnesota Code (MC) classification system for electrocardiographic findings using “x” to assign sub-classification criterion [24, 25]. Major ECG abnormalities were defined by the presence of at least one of the following: old (MC 1.1.x or 1.2.x) or possible myocardial infarction (1.3.x and 4.1.x, 4.2, 5.1, or 5.2), complete intraventricular blocks (MC 7.1, 7.2, 7.4, or 7.8), frequent supraventricular or ventricular premature beats (MC 8.1.x, except 8.1.4), major isolated ST segment or T-wave abnormalities (MC 4.1.x, 4.2, 5.1 or 5.2), atrial fibrillation or flutter or supraventricular tachycardia (MC 8.3.x. or 8.4.2), other major arrhythmias (MC 8.2.x, except 8.2.1), major atrioventricular conduction abnormalities or pacemaker use (MC 6.1, 6.2.x, 6.4, 6.8, 8.6.1 or 8.6.2), major QTi prolongation (> 115%) and left ventricular hypertrophy (LVH) (MC 3.1 together with [4.1.x, 4.2, 5.1, or 5.2]). Further details can be seen elsewhere [26].

### Biomarkers (cytokines and chemokines)

Blood samples for the measurement of cytokines and chemokines were collected at the baseline survey in early morning. The Cytometric Bead Array assay (CBA immunoassay kit; Becton, Dickinson and Company BD Life Sciences - Biosciences, San Jose, CA, USA) was used for quantitative determination of serum cytokines (Human Inflammatory kit) and chemokines (Human Chemokines kit) according to manufacturer (Becton, Dickinson and Company BD Life Sciences - Biosciences, San Jose, CA, USA). The Inflammatory CBA kit comprises micro-beads coupled to monoclonal antibodies (MoAb) against the following cytokines IL-6, IL-12, TNF, IL-10, and IL-1β, and the chemokine CBA kit detects CXCL8, CCL2, CXCL9, CCL5 and CXCL10. A second fluorescently labelled phycoerythrin (PE)-anti-cytokine antibody was used and the concentration of the individual cytokines was indicated by fluorescence intensity. Data was acquired using a FACSVerse flow cytometer (Becton Dickinson, USA). Sample analysis was performed with BD FCAP Array 3.0 software (Becton, Dickinson and Company BD Life Sciences - Biosciences, San Jose, CA, USA). The coefficients of variation for intra and inter-assays were 5–10% and 7–12%, respectively.

### Potential confounding variables

Potential confounding variables in our analysis included socio-demographic characteristics (age, gender, schooling and family income), lifestyle (current smoking, physical activity and alcohol consumption), chronic diseases and medication use (digoxin and anti-inflammatory medication). Monthly family income per capita was divided into tertiles (< USD 90.00 was the lowest tertile). Current smokers were those who had smoked at least 100 cigarettes during their lifetimes and who were still smokers. Physical activity was calculated based on the level of oxygen consumed for 25 physical activities in the previous 3 months, as described elsewhere [27]. Sedentary individuals were considered to be those whose energy expenditure was less than 450 metabolic equivalent tasks, which corresponds to at least 150 min per week of moderately to vigorously physical activity [27]. Alcohol consumption was defined by consumption of 14 doses or more per week in the previous 12 months. Chronic diseases considered in our analysis were: hypertension (systolic blood pressure ≥ 140 mmHg and/or diastolic blood pressure ≥ 90 mmHg and/or treatment); diabetes (fasting blood glucose ≥ 7.0 mmol/L and/or treatment); arthritis and myocardial infarction as defined by a previous medical diagnosis for the condition; stroke, using a standard protocol [28]; total cholesterol (mmol/L), triglycerides (mmol/L), body mass index (kg/m^2^) and creatinine level (μmol/L) (as continuous variables); depressive symptoms, which were calculated using the 12-item version of the General Health Questionnaire; a score ≥ 5 was considered the cut-off for defining exposure status, as recommended for the study population [29].

Fasting total cholesterol, triglycerides, glucose and creatinine were determined by using standard enzymatic methods (Merck, Darmstadt, Germany). Body mass index (BMI) was defined by weight divided by height squared (kg/m^2^). Current medication use was ascertained during the home interview by reviewing prescriptions and/or medication packaging. Blood Pressure (BP) was measured 30 or more minutes after the last caffeine intake or cigarette smoked. Three measures were taken after 5 min of initial rest and subsequently at 2-min intervals.

### Statistical analysis

The outcome variables were ten biomarkers (cytokines and chemokines), as previously described. Based on their distribution, IL-6, CXCL10, CCL2, CXCL9, CCL5 and CXCL8 were dichotomized as above or below the median (coded 1 and 0, respectively). Given their lower values, IL-12, TNF, IL-10 and IL-1β were dichotomized as the lowest detectable value vs. non-detectable (coded as 1 and 0, respectively). The exposure variables were *T. cruzi* infection and ECG abnormality, which were categorized as follows: seronegative non-infected (NI = reference category), infected without major ECG abnormalities (CH (N) ECG) and infected with major ECG abnormality (CH (Ab) ECG).

Pearson’s chi square test was used in bivariate analyses to assess the statistical significance of differences across the three groups described above. Multivariate analyses were conducted for the biomarkers that showed significant association (*P* <  0.05) in the previous analysis, considering adjustment for all potential confounders included in the present study. For this analysis, the biomarkers were dichotomized or divided in tertiles, according to their distribution. The adjusted odds ratio and 95% confidence intervals were estimated for binary logistic or multinomial logistic regression. The predicted probability was then estimated and plotted for each biomarker, considering the possible interaction between age and *T. cruzi* infection/ECG abnormality.

Since there was no evidence of interaction with sex (*P* value for interaction > 0.05 for all), the analyses were carried out for both men and women with sex included as a covariate. All analyses used Stata version 13 (StataCorp LLP, College Station, TX).

## Results

### Baseline characteristics of the study participants

Of the 1606 baseline cohort participants, 1284 had complete information for all study variables and were included in the current analysis. The baseline characteristics of the study participants are shown in Table 1. Among study participants, the mean age was 68.8 years with women predominating (61.9%). Statistically significant differences (*P* < 0.05) across the study groups (seronegative-NI, seropositive with and without electrocardiogram ECG abnormalities) were found for age, gender, educational level, family income, depressive symptoms, body mass index, serum creatinine level and digoxin use (Table 1).

**Table 1:** Baseline characteristics of study participants and by *Trypanosoma cruzi* infection serological status

Characteristics	TOTAL (*n* = 1284)	NI (*n* = 802)	CH (N) ECG (*n* = 210)	CH (Ab) ECG (*n* = 272)	*P* value
Age in years, mean (*SD*)	68.8 (6.9)	68.5 (7.0)	68.0 (6.6)	70.4 (6.9)	**< 0.001**
Female, %	61.9	57.4	70.5	68.8	**< 0.001**
Schooling < 4 years, %	63.7	51.4	82.4	85.7	**< 0.001**
Family income^a^, %	27.4	24.8	31.9	31.6	**0.026**
Current smoking^b^, %	17.0	17.5	15.7	16.5	0.817
Alcohol consumption^c^, %	2.2	2.0	1.9	2.9	0.624
Sedentary lifestyle^d^, %	26.7	25.4	29.1	28.7	0.409
Arthritis^e^, %	31.9	32.2	31.4	31.3	0.951
Diabetes^f^, %	14.8	17.3	9.1	11.8	0.003
Myocardial infarction, %	4.7	4.6	3.3	5.9	0.418
Stroke^e^, %	3.4	2.6	3.3	5.5	0.072
Hypertension^g^, %	61.5	61.6	57.1	64.3	0.271
Depressive symptoms^h^, %	37.6	33.2	41.4	47.8	**< 0.001**
Total Cholesterol (mmol/L), mean (*SD*)	6.0 (1.3)	6.0 (1.2)	6.1 (1.3)	6.0 (1.3)	0.992
Triglycerides (mmol/L), mean (*SD*)	1.7 (1.1)	1.8 (1.2)	1.7 (1.1)	1.6 (1.0)	0.181
Body mass index, mean (*SD*)	25.2 (5.0)	25.6 (4.9)	25.0 (5.0)	24.0 (5.2)	**< 0.001**
Creatinine (μmol/L), mean (*SD*)	80.0 (30.0)	80.0 (30.0)	70.0 (20.0)	80.0 (20.0)	**0.040**
Digoxin use, %	14.4	10.4	17.1	24.3	**< 0.001**
Anti-inflammatory medication, %	16.2	15.8	18.6	15.4	0.587

CH: Chagas disease patients; NI: Non-infected subjects; N: Normal; Ab: Abnormal; ECG: Electrocardiogram; *SD*: standard deviation.

^a^Monthly family income per capita (lowest tertile); ^b^Current smoking (who had smoked at least 100 cigarettes during their lifetimes and who were still smokers); ^c^Alcohol consumption in previous 12 months (> 14 doses per week); ^d^Sedentary Lifestyle (< 150 min of physical activity per week); ^e^Arthritis and Stroke (medical diagnosis); ^f^ Diabetes (fasting blood glucose ≥ 7.0 mmol/L and/or treatment); ^g^Hypertension (systolic blood pressure ≥ 140 mmHg and/or diastolic blood pressure ≥ 90 mmHg and/or treatment); ^h^Depressive symptoms (General Health Questionnaire score ≥ 5); *P* value from ANOVA for continuous variables and chi square test for categorical variables

### Bivariate association between *T. cruzi *infection and serum biomarker levels

Bivariate analysis between ten biomarkers and the study groups above mentioned is demonstrated at Table 2. Statistically significant associations were found for serum concentration (pg/ml) of CXCL9, CXCL10, and CCL5 above or below the median levels and also for serum detectable levels of IL-1β.

**Table 2:** Bivariate association between *Trypanosoma cruzi* infection and serum chemokines/cytokines levels above the median or detectable values

Biomarkers*	Percentage above the global median			*P* value
	NI	CH (N) ECG	CH (Ab) ECG	
Chemokines (cut-offs)				
CCL2 (≥ 50.24 pg/ml)	33.3	31.4	33.8	0.843
CXCL9 (≥ 3485.80 pg/ml)	**21.9**	**47.6**	**54.4**	**< 0.001**
CXCL10 (≥ 4014.77 pg/ml)	**23.8**	**46.2**	**50.0**	**< 0.001**
CCL5 (< 630.37 pg/ml)	**36.4**	**22.9**	31.3	**< 0.001**
CXCL8 (≥ 4.41 pg/ml)	33.8	30.0	32.0	0.553
Cytokines (cut-offs)				
IL-1β (≥ 0.01 pg/ml)	**12.2**	**41.9**	**37.1**	**< 0.001**
IL-6 (≥ 1.65 pg/ml)	32.4	29.5	37.5	0.153
TNF (≥ 0.01 pg/ml)	16.8	17.6	18.0	0.892
IL-12 (≥ 0.01 pg/ml)	7.6	6.7	8.8	0.668
IL-10 (≥ 0.07 pg/ml)	32.8	33.3	39.3	0.138

*CH* Chagas disease patients, *NI* Non-infected subjects, *N* Normal, *Ab* Abnormal, *ECG* Electrocardiogram, *TNF* Tumor necrosis factor, *IL-6* Interleukin 6, *IL-1β* Interleukin 1 beta, *IL-10* Interleukin 10, *IL-12* Interleukin 12, *CXCL9* C-X-C motif chemokine ligand-9, *CCL5* C-C motif chemokine ligand 5, *CXCL10* C-X-C motif chemokine ligand-10, *CXCL8* C-X-C motif ligand 8, *CCL2* C-C motif chemokine ligand 2

*Cut-offs points above the median except for IL-1β, TNF, IL-12 and IL-10 (detectable values). *P* value: from chi-square test. Altered levels according to cut-offs. *P* value of chi-square test

### Multivariate analysis of inflammatory markers and *T. cruzi* infection

The statistically significant results of the multivariate analysis of the association between *Trypanosoma cruzi* infection and biomarkers are presented at Table 3. After adjustments for potential confounders, positive and independent positive associations were found from the biomarkers evaluated showing that the groups CH (N) ECG and CH (Ab) ECG presented an odds ratio (*OR*) of 4.14 and 3.02 for CXCL9; 2.26 and 2.95 for CXCL10; 2.65 and 1.57 for CCL5; 5.02 and 3.94 for the cytokine IL-1β, respectively, suggesting significant differences in the levels of cytokines/chemokines among the groups studied.

**Table 3:** Multivariate analysis of the association between inflammatory markers and *Trypanosoma cruzi* infection

Serum levels in tertiles	Infection status – odds ratio (95% confidence interval) *		
	Negative	Positive	
		CH(N)ECG	CH (Ab)ECG
CXCL9 (pg/ml)			
Intermediate (1569.1–3483.0)	1.00	1.25 (0.79–2.00)	0.68 (0.45–1.04)
Highest (> 3483.0)	1.00	4.14 (2.65–6.48)	3.02 (2.05–4.45)
CXCL10 (pg/ml)			
Intermediate (2352.8–4018.0)	1.00	0.90 (0.59–1.37)	1.17 (0.78–1.75)
Highest (> 4018.0)	1.00	2.26 (1.52–3.37)	2.95 (2.00–4.34)
CCL5 (pg/ml)			
Intermediate (644.7–1263.6)	1.00	1.93 (1.26–2.95)	1.40 (0.96–2.03)
Lowest (< 644.7)	1.00	2.65 (1.75–4.02)	1.57 (1.08–2.28)
IL1-β (pg/ml)			
Detectable (> 0.01)	1.00	5.02 (3.46–7.27)	3.94 (2.76–5.63)

*CH* Chagas disease patients, *N* Normal, *Ab* Abnormal, *ECG* Electrocardiogram, *CXCL9* C-X-C motif chemokine ligand-9, *CCL5* C-C motif chemokine ligand 5, *CXCL10* C-X-C motif chemokine ligand-10

*Estimated by multinomial^1^ or binomial logistic^2^ regression and adjusted for all variables listed in Table 1. First tertile as a reference category for CXCL10 and CXCL9; third tertile is a reference category for CCL5

### Predicted probability for variation in serum biomarker levels along the age continuum

**Fig. 1:**
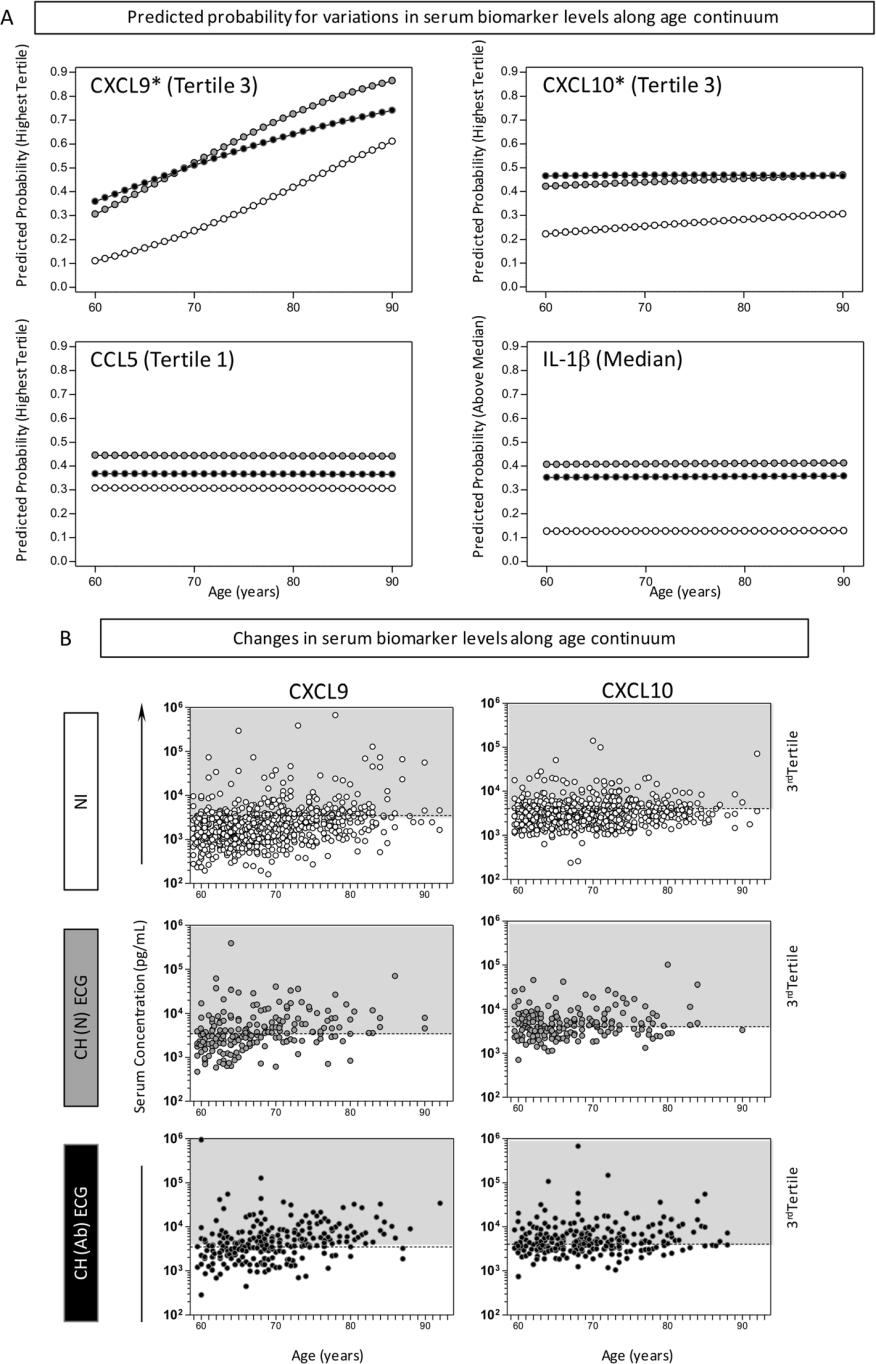
Predicted probability for variations in serum biomarker levels along age continuum. **a** Predicted probability of high producers of serum biomarkers (CXCL9, CXCL10, CCL5 and IL-1β) along ageing continuum for Chagas disease subgroups CH(N) ECG (); CH (Ab) ECG () and non-infected controls NI (). **b** Scattering distribution of changes in CXCL9 and CXCL10 levels along age continuum. The gray background underscored the 3rd Tertile to highlight the higher proportion of CH (Ab) ECG subjects above the threshold. The modeled system employed is described in methods section. IL-1β: Interleukin 1 beta CXCL9: C-X-C motif chemokine ligand-9; CCL5: C-C motif chemokine ligand 5; CXCL10: C-X-C motif chemokine ligand-10; ECG: Electrocardiogram; NI: Seronegative non-infected; CH (N) ECG: infected without major ECG abnormalities; CH (Ab) ECG: infected with major ECG abnormality

The results of the predicted probability of changes in serum chemokines/cytokines levels (CXCL9, CXCL10, CCL5 and IL-1-β) along the age continuum revealed that CXCL9 and CXCL10 levels from all groups increased along the age continuum, suggesting a strong association between the production of these biomarkers and elderly patients. No association was observed between levels of IL-1β and CCL5 and age (Fig. 1). Moreover, Fig. 1b clearly shows that the groups CH(N) ECG and CH (Ab) ECG had higher numbers of individuals with high levels of CXCL9 and CXCL10 and that this is associated with age, suggesting changes in serum levels of these biomarkers during aging.

Regarding potential confounding variables, it is important to mention that none of the cohort participants had a history of anti-*T. cruzi* etiological treatment on entry into the cohort, and none received treatment during the period in which patients were followed [21]. However, the lack of information about treatment for other infectious diseases or about conditions of re-infection could be residual confounders for the associations between *T. cruzi* infection and inflammatory markers.

## Discussion

This large population-based cohort study provided unique information about the characteristics of the study participants and *T. cruzi* infection. Our data showed significant and positive associations between *T. cruzi* infection and female gender, body mass index, digoxin use and depression. Moreover, our data showed a strong association between low schooling level (an indicator of socioeconomic status) and *T. cruzi* infection.

Previous studies have shown that inflammatory cytokines and chemokines play a central role in *T. cruzi* infection [6–8]. Inflammation is a key mechanism in the initiation, progression and clinical manifestations of Chagas heart disease in which the presence of *T. cruzi* or its antigens triggers an uncontrolled humoral and / or cellular immune response. Our data showed that infected Chagas patients presented significantly increased levels of the cytokine IL-1β and the chemokines CXCL10 and CXCL9, and decreased levels of CCL5, when compared to non-infected individuals (Tables 2 and 3).

Chagas disease is a lifelong infection in humans that can be manifested many years after initial infection. Moreover, inflammation disproportional to parasite load characterizes chronic myocardial lesions in chagasic individuals. It has been reported that plasma levels of IL-1β were increased in the early phase of acute myocardial infarction and that the increase was observed prior to the peak plasma levels of brain natriuretic peptide/BNP [30, 31]. Also, the expression of IL-1β mRNA has been observed in the infarct region of the heart [32]. Thus, these authors hypothesized that IL-1β might induce BNP secretion and cardiac hypertrophy in acute myocardial infarction or heart failure [33]. Additionally, Sousa et al., [34] evaluated plasma cytokine levels in well defined clinical polar groups of chronic Chagas patients and observed that inflammatory cytokine expression, including IL-1β, was highest in the cardiac (CARD) group.

Further studies about hypertrophic response in *T. cruzi*-infected cardiac myocytes identified the proinflammatory cytokine IL-1β as an important mediator. These authors demonstrated, by the addition of a specific cytokine trap to infected cardiac myocyte cultures, that blocking the activity of IL-1β leads to a significant reduction of the overall hypertrophic response. Indeed, IL-1β, which is rapidly induced in response to *T. cruzi*, promotes cardiomyocyte hypertrophy early in the infective process and may contribute to the maintenance of cardiomyocyte function during establishment of *T. cruzi* infection in the heart [35, 36]. Medeiros et al., [37] demonstrated that patients with the cardiac clinical form had increased IL-1β associated with metalloproteinase (MMP-9) and alternative caspase-1-independent pathway. These findings, together with our data that showed high levels of IL-1β in Chagas patients, suggest that this cytokine has an important role in disease progression.

Few studies have evaluated the relevance of chemokines for *T. cruzi* infection in humans but studies on experimental infection showed that during chronic infection of C57BL/6 mice with the Colombian strain of *T. cruzi*, as in the acute experiments, there was a clear correlation between the migration of leukocyte subsets and the expression of particular chemokines [38, 39]. Importantly, CXCL10, CXCL9 and CCL5 were produced throughout the course of infection (until day 120), even when tissue parasitism was low [38]. Also, CXCL10 and CCL5 were found to be among the dominant cytokines expressed in situ during acute experimental infection and chronic phase of *T. cruzi*-elicited myocarditis contributing to intense recruitment of activated T cells [40].

Our data corroborate these findings, except for the low levels of CCL5 presented by CH(N) ECG patients. Marino et al. [15] demonstrated that most of the inflammatory cells invading the heart tissue during the early phase of *T. cruzi* infection of C3H/HeJ mice were CD8^+^ T cells and that they expressed CCR5, a CCL5 receptor. In humans, previous studies found increased percentages of effector CD4^+^ T cells and central memory CD4^+^ and CD8^+^ T cells expressing CCR5 in patients with structural cardiopathy, but normal global ventricular function and no symptoms of chronic heart failure [41]. However, a recent work from Luz et al. [42] with chronic human patients did not show any difference in the levels of CCL5 in plasma samples of Chagas patients and controls. In fact, Chagas patients presented lower levels than controls (8829.0 pg/μl vs 9259.0 pg/μl; *P* = 0.052). This finding is corroborated by our results indicating that CCL5 is more important in situ instead of systemic response.

Interestingly, Crema et al. [43] also showed that CXCL9 levels were significantly higher in chagasic patients with megaesophagus compared to individuals without the disease. In addition, studies have shown significantly higher levels of CXCL10 in patients with chronic Chagas disease when compared to controls, suggesting that CXCL10 may contribute to inflammation and consequent tissue injury in the cardiac form of CD [42, 44]. Additionally, our results showed that levels of CXCL9 and CXCL10 for Chagas patients increased along age continuum, suggesting an association between these chemokines and elderly patients. Furthermore, CXCL10 has been considered a novel biomarker for severity of parasitic diseases [45].

Also, Cunha-Neto et al. [18] showed that IFN-γ-dependent chemokines, such as CCL5, CXCL9 and CXCL10, may increase the chemotactic signal and cause migration of more CCR5^+^ CXCR3^+^ T lymphocytes to the myocardium. Recent studies by this group showed that genetic variation in chemokines genes could be associated with CCC development. These authors demonstrated that polymorphisms in CXCL9, CXCL10 and CCR5 were associated with differential risk of progression to the more severe form of CCC, suggesting that such chemokines may be master regulators of myocardial inflammatory cell migration, perhaps affecting clinical progression to severe CCC [46].

This study may present limitations inherent of observational investigation and should be further validated by long-term longitudinal follow up. Since Bambui city has been considered a geographical area endemic for Chagas disease for many decades, it is possible that most patients acquired the infection at childhood and, therefore, these changes in serum biomarker profiles may be the result of a multifactorial process that includes both the ageing process and the time elapsed since *T. cruzi* primo-infection.

## Conclusions

Our results provide new evidence supporting the importance of the cytokine IL-1β and the chemokines CXCL9, CXCL10 and CCL5 in *T. cruzi* chronic infection in humans. Levels of CXCL9 and CXCL10 for Chagas patients increase along the age continuum, suggesting a strong association between the production of these chemokines and elderly patients. Moreover, the association of two or more biomarkers, especially CCL5 and IL1-β, may indicate the progression of clinical symptoms in Chagas patients. Thus, the functional role of particular chemokines in host resistance to infection and in the pathogenesis of Chagas disease remains to be investigated.

## Data Availability

The datasets used and/or analyzed during the current study are available from the corresponding author on reasonable request.

## Abbreviations

CD: Chagas disease
CCC: Chagas disease cardiomyopathy
IFN-γ: Interferon gamma
TNF: Tumor necrosis factor
IL-6: Interleukin 6
IL-1β: Interleukin 1 beta
IL-4: Interleukin 4
IL-10: Interleukin 10
IL-12: Interleukin 12
CXCR3: C-X-C motif chemokine receptor 3
CCR5: C-C chemokine receptor type 5
CXCL9: C-X-C motif chemokine ligand-9
CCL5: C-C motif chemokine ligand 5
CXCL10: C-X-C motif chemokine ligand-10
ECG: Electrocardiogram
MC: Minnesota code
LVH: Left ventricular hypertrophy
CBA: Cytometric bead array assay
CXCL8: C-X-C motif ligand 8
CCL2: C-C motif chemokine ligand 2
MoAb: Monoclonal antibody
BMI: Body mass index
BP: Blood pressure
NI: Seronegative non-infected
CH (N) ECG: Infected without major ECG abnormalities
CH (Ab) ECG: Infected with major ECG abnormality
BNP: Brain natriuretic peptide
MMP-9: Metalloproteinase 9
CD4+ T cells: CD4 T lymphocytes
CD8+ T cells: CD8 T lymphocytes
